# Quality of YouTube Videos Related to Colorectal Cancer Screening

**DOI:** 10.7759/cureus.33684

**Published:** 2023-01-12

**Authors:** Aaron Kahlam, Suraj Pai, Jasneel Kahlam, Sushil Ahlawat

**Affiliations:** 1 Internal Medicine, Rutgers New Jersey Medical School, Newark, USA; 2 Internal Medicine, University of Michigan School of Medicine, Ann Arbor, USA; 3 Internal Medicine, New York Institute of Technology College of Osteopathic Medicine, Old Westbury, USA; 4 Gastroenterology and Hepatology, Rutgers University, Newark, USA

**Keywords:** adult gastroenterology, internet technologies, bowel cancer screening, colorectal cancer, patient education

## Abstract

Introduction

YouTube, an unregulated video-sharing website, is the second most visited website on the internet. As more patients turn to the internet for information about colon cancer screening, it is important to understand what they are consuming online. Our goal was to evaluate YouTube videos about colon cancer screening to better understand the information patients are accessing.

Methods

We searched YouTube on October 28, 2020, using the following search terms sorted by relevance and view count: colonoscopy, colon cancer screening, virtual colonoscopy, colonoscopy alternatives, and cologuard. Videos longer than 10 minutes, not in English, and duplicates were excluded. Three evaluators graded each video using the DISCERN criteria. Numerical data were averaged into a composite score. Two-sided t-tests and one-way ANOVA tests were used to compare mean ratings between groups.

Results

Fifty videos were analyzed, with a total of 23,148,938 views, averaging 462,979 views per video. The average overall rating was 3.16/5. There was no difference between search methods, search terms, or presence of a physician. The average ratings for videos with gastroenterologists (3.08), other physicians (3.35), and non-physicians (3.09) were not significantly different. Videos without physicians had more views on average (1,148,677) compared to videos with gastroenterologists (157,846, p=0.013) or other physicians (35,730, p=0.013).

Conclusion

YouTube videos related to colon cancer screening were of good quality regardless of search terms, search methods, or presence of a physician. However, videos without physicians were viewed more frequently. Physicians should continue making videos that address deficits while increasing viewership.

## Introduction

Colorectal cancer (CRC) is the third most common cancer and the second most common cause of death from cancer in the United States and the world [1,2]. While there are a variety of screening options, including colonoscopy, virtual colonoscopy, and fecal immunohistochemical testing (FIT) [3-5], only 68.8% of adults aged 50-75 years were up to date with their colon cancer screening according to the CDC in 2018 [6]. This may be due to a variety of factors, including misinformation [7-9] and anxiety or embarrassment [9,10]. In seeking more information, many patients may turn to the internet to learn more about screening and to alleviate any anxiety they may have.

It is well known that a large percentage of patients utilize the internet for health information [11,12]. YouTube^TM^ (San Bruno, CA: Google Inc.) is a video-sharing website that receives two billion viewers each month, making it the second most visited website in the world and likely an important source of information for patients [13]. As a video-sharing website, it contains a wealth of information on a wide variety of topics with little regulation. However, there are relatively few studies looking at the quality of information on YouTube regarding this important topic. One recent study has shown that YouTube is a poor source of information related to colonoscopies [14], while another showed that it was potentially a useful tool for education on CRC [15]. Other studies have looked at the effectiveness of YouTube videos for bowel prep instruction [16,17]. However, differing search methods, analytic tools, and exclusion criteria make it difficult to compare studies. Thus, further research into content published on YouTube using a standardized and validated questionnaire would help physicians better understand and compare content on different aspects of CRC available on YouTube.

In this analysis, we aimed to expand on previous research related to YouTube videos about CRC and screening by using a validated questionnaire. We also focus on highlighting aspects of CRC screening that are lacking in videos so that physicians are aware of what deficiencies in knowledge, patients who elect to search online, may have. Finally, we compare content created by physicians to that created by non-physicians to understand what type of content patients prefer to view online.

## Materials and methods

**Figure 1:**
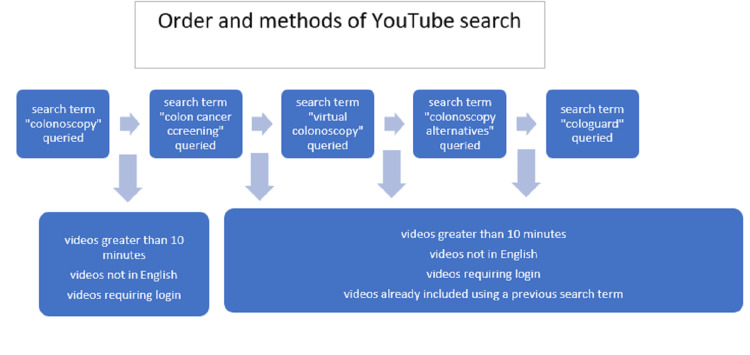
Flow chart demonstrating the sequence of searches and exclusion criteria. Terms were searched from left to right, and videos included in a previous search were not included in a subsequent search.

The search terms “colonoscopy,” “colon cancer screening,” “virtual colonoscopy,” “colonoscopy alternatives,” and “cologuard” were queried in YouTube’s search engine on October 10, 2020, and sorted by relevance and views. This search was done using Google Chrome and in “incognito” mode so that previous searches did not impact future results. Figure 1 demonstrates the sequence of searches as well as the exclusion criteria used. The top five videos that met inclusion criteria for relevance and view count were chosen for a total of 10 videos per search term. Videos that were not in English, required the user to log in, or were over 10 minutes in duration were not assessed. Repeat videos were only assessed once. The videos were rated using the DISCERN tool, a validated survey created by Oxford University and the British Library through a collaboration of healthcare professionals, laypeople, and information experts [18,19]. The survey contains 15 questions, with eight assessing the reliability and seven assessing the information with a final question asking the evaluator to rate the overall quality (Table 1). Five questions from the questionnaire were excluded because they were not relevant to the content of the video (Table 1). Each question was rated on a scale of 1-5 with 5 being the most complete and 1 being the least complete information. For the question “is it clear what sources of information were used,” a United States licensed physician speaking with no citation was defined as a three on the scale. Each video was viewed by three evaluators who were blinded to each other’s ratings. Two of the raters were fourth-year medical students who had completed pre-clinical education in gastroenterology and spent a month on a gastroenterology rotation. The third reviewer was a second-year medical student who had completed pre-clinical studies in gastroenterology but had no clinical rotation. All three evaluators had background knowledge of screening options and guidelines for colon cancer. The numerical ratings from the evaluators were then averaged into one composite score per video for the analysis.

**Table 1:** List of DISCERN questions. *Questions not used in this analysis.

Are the aims clear?*
Does it achieve its aims?
Is it relevant?
Is it clear what sources of information were used to compile the publication?
Is it clear when the information used or reported in the publication was produced?*
Is it balanced or unbiased?
Does it provide details about additional sources of support and information?*
Does it refer to areas of uncertainty?
Does it describe how each treatment works?
Does it describe the benefit of each treatment?
Does it describe the risks of each treatment?
Does it describe what would happen if no treatment was used?*
Does it describe how the treatment choices affect overall quality of life?*
Is it clear that there may be more than one possible treatment choice?
Does it provide support for shared decision-making?
Based on the answers to all of the above questions, rate the overall quality of the publication as a source of information about treatment choices.

Along with the DISCERN questions, data was recorded about number of views, length, number of subscribers, likes and dislikes, category (i.e., patient education, advertisement, entertainment, personal/patient experience or operative), and the credentials of the author (i.e., physician, specialty). Disagreements in these variables were resolved using consensus. Simple descriptive statistics were used, and a two-sided t-test was used to determine significance between videos. A one-way ANOVA test was used to compare mean ratings between different types of physicians (gastroenterologist, other, or none) and number of views between the three groups. All statistical analyses were performed using IBM SPSS version 24 (Armonk, NY: IBM Corp.).

## Results

After performing the search, 76 videos were found. Ten videos were longer than 10 minutes, four required the user to sign in, 11 were repeats, and one was in another language, which left 50 videos to be assessed. The videos had a total of 23,148,938 views, 95,583 likes, and 7,487 dislikes. Median views, likes, dislikes, and length with interquartile ranges are shown below (Table 2). Average ratings for each DISCERN question are shown in Table 3. The average overall rating for all videos was 3.16/5. Most videos were rated low for their discussion of risks (1.83/5) and shared decision-making (2.38/5) though they were rated highly in discussing how the screening methods worked (3.87/5).

**Table 2:** Median views, likes, dislikes, and length with interquartile rage.

Value	Median (rounded)	Interquartile range (25-75%)
Views	35,828	5,313-163,258
Likes	78	10-838
Dislikes	11.5	1-53.5
Length (seconds)	188	141-400

**Table 3:** Average rating by DISCERN question.

DISCERN question (abbreviated)	Average	Standard deviation
Achieving aims	3.65	0.70
Relevance	3.74	0.82
Sources	2.85	0.66
Bias	3.64	0.81
Areas of uncertainty	2.93	0.86
Explanation of screening	3.87	1.06
Discussing benefits	3.44	0.98
Discussing risks	1.83	1.14
Discussing options	3.38	1.64
Shared decision making	2.39	0.82
Overall rating	3.16	0.80

The breakdown of videos regarding specialty of physician featured was 20/50 featuring a gastroenterologist and 13/50 featuring a physician from another specialty (including oncology, pathology, urology, radiology, and family medicine). The remaining 17 videos did not have any physician as the main contributor. There was no significant difference in overall rating between videos sorted by relevance (3.15) and view count (3.17), p=0.934; or videos with a board-certified physician (3.19) vs. videos without a board-certified physician (3.10), p=0.734. Additionally, there was no significant difference between different search terms (Table 4). By specialty, there was no significant difference in rating between gastroenterologists (3.08, reference), defined as physicians board-certified in gastroenterology; non-gastroenterologist physicians (3.36, p=0.817), defined as physicians board-certified in a field other than gastroenterology; and non-physicians (3.10, p=1.00). However, there was a significant difference in views. Non-physician videos had an average of 1,148,677 views (reference), while videos featuring gastroenterologists had an average of 157,846 views (p=0.013), and videos featuring other physicians had an average of 35,730 views (p=0.013).

**Table 4:** Results of ANOVA comparing averages by each search term. Ref. delineates reference, in that all terms were compared to the search term colonoscopy.

Search term	Average overall rating	p-Value
Colonoscopy	3.00	Ref.
Colon cancer screening	3.17	0.999
Colonoscopy alternatives	3.40	0.937
Virtual colonoscopy	2.87	1.000
Cologuard	3.37	0.956

## Discussion

In this study, we quantitatively analyzed highly accessed YouTube videos related to colon cancer screening across five search terms and two search methods using the DISCERN criteria. Overall, the quality of the videos was good, and these results were consistent across different search terms, search methods, and contributors. Videos tended to not discuss or not go into detail about risks associated with screening, though they were effective in explaining how the screening method worked. Finally, despite being of equal quality, videos with non-physician contributors had 10 times more views than those that had a physician contributing. This data is consistent with Sahin et al. who showed that videos related to colon cancer were suitable for some patients but not a complete source of information and that videos with lower ratings, which typically did not feature a physician, had more views [15].

Despite the encouraging results, videos were consistently deficient in talking about risks of procedures, while also being deficient in discussing and encouraging shared decision making as shown by their relatively low ratings. Risks are often difficult to discuss for physicians, who often believe that colonoscopy is the best option for their patients, leading them to either not address them or not spend enough time on them [20,21]. This may be exaggerated in the video format by the lack of a personal relationship with the patient. Videos also tended to imply risk, without going into details about the epidemiology or potential consequences of those risks. We hypothesize that many of the videos are designed to encourage screening, and, therefore, might be reluctant to provide information that may dissuade patients from being screened. Videos tended to perform well in explaining how the screening method works and the benefits of CRC screening, which further supports our hypothesis. Though videos promoted screening, bias was generally mitigated through use of evidence-based data, epidemiological statistics, and neutral language. Nearly 70% of videos contained a physician, which helped to limit medical misinformation and maintain an objective viewpoint.

Another concerning finding from our analysis was the fact that videos not featuring a physician in any specialty had around 10 times as many views as those featuring a gastroenterologist even though 40% of videos did feature one, which was consistent with several other studies [15,22-24]. This discrepancy suggests a disconnection between content that physicians are producing and what patients are viewing. In addition to measuring popularity of a video, views are an important metric in YouTube’s search engine, as relevant videos with more views will appear higher. Thus, physicians who are creating content with the goal of educating patients should consider not only the information they wish to convey but also the way in which they convey that information. For example, previous studies have shown that videos three to five minutes long that tend to evoke strong emotions and relate to the target audience are more likely to be viewed [25-27]. An example from our analysis was a video titled “what happens during and after a colonoscopy” created by a channel titled "You and Colonoscopy" had over 4.5 million views. This video was just over five minutes long and contained simple diagrams and language to explain what happens during a colonoscopy and why it is important. Simplifying videos and incorporating tools, such as simple diagrams, humor, and patient testimonials, may help physicians better connect online with patients seeking more information about CRC screening.

Our study does have some important limitations to consider. We used strict inclusion criteria focusing on the length and language used in the videos in an effort to limit videos to those that were most likely to be viewed. Therefore, we cannot generalize our findings and conclusions to videos that were longer than 10 minutes or made in languages other than English. YouTube is also a rapidly evolving website, we queried its engine with a specific set of search terms in a single day and it is possible that more comprehensive material has been published in the time since. Finally, our panel was composed of individuals with varying degrees of medical backgrounds, and, therefore it is difficult to ascertain how evaluators with non-medical backgrounds or those with poor health literacy would rate the videos. Future studies may want to include evaluators who do not have a medical background to gain a better understanding of their perspective.

As technology advances and more information becomes easily accessible, it is inevitable that more patients will turn to the internet for health information. Websites such as YouTube and Twitter are some of the most visited websites on the Internet, with 34.6 billion and 6.6 billion respective visitors monthly [28]. Unsurprisingly, patients often utilize these and other platforms for medical education. While it may be difficult to control medical misinformation, physicians can use the results of our analysis to better understand what their patients are viewing online and how to supplement that information to ensure they are making informed decisions. Additionally, providers looking to create content for patients should be aware of the discrepancy in views between videos featuring a physician compared to those that do not so that they can tailor their videos to appeal to a wide variety of patients online.

## Conclusions

In conclusion, we found that videos on YouTube related to colorectal cancer screening were of good quality but had some notable shortcomings. These findings were consistent across search terms, search methods, and contributors. Worryingly, videos featuring physicians had about 10 times fewer views than those not featuring a physician. Physicians should be aware of the deficiencies found in online material related to CRC screening and be prepared to address these shortcomings in the clinic. Furthermore, public health organizations featuring a physician to promote CRC screening should try to utilize social networks, comedy, and other tools to broaden their audience.
